# Minimal inhibitory concentration distributions and epidemiological cutoff values of five antifungal agents against *Sporothrix brasiliensis*

**DOI:** 10.1590/0074-02760160527

**Published:** 2017-05

**Authors:** Rodrigo Almeida-Paes, Fábio Brito-Santos, Maria Helena Galdino Figueiredo-Carvalho, Ana Caroline Sá Machado, Manoel Marques Evangelista Oliveira, Sandro Antonio Pereira, Maria Clara Gutierrez-Galhardo, Rosely Maria Zancopé-Oliveira

**Affiliations:** 1 Fundação Oswaldo CruzFundação Oswaldo Cruz-FiocruzInstituto Nacional de Infectologia Evandro ChagasLaboratório de MicologiaRio de JaneiroRJBrasilFundação Oswaldo Cruz-Fiocruz, Instituto Nacional de Infectologia Evandro Chagas, Laboratório de Micologia, Rio de Janeiro, RJ, Brasil; 2 Fundação Oswaldo CruzFundação Oswaldo Cruz-FiocruzInstituto Nacional de Infectologia Evandro ChagasLaboratório de Pesquisa Clínica em Dermatozoonoses em Animais DomésticosRio de JaneiroRJBrasilFundação Oswaldo Cruz-Fiocruz, Instituto Nacional de Infectologia Evandro Chagas, Laboratório de Pesquisa Clínica em Dermatozoonoses em Animais Domésticos, Rio de Janeiro, RJ, Brasil; 3 Fundação Oswaldo CruzFundação Oswaldo Cruz-FiocruzInstituto Nacional de Infectologia Evandro ChagasLaboratório de Pesquisa Clínica em Dermatologia InfecciosaRio de JaneiroRJBrasilFundação Oswaldo Cruz-Fiocruz, Instituto Nacional de Infectologia Evandro Chagas, Laboratório de Pesquisa Clínica em Dermatologia Infecciosa, Rio de Janeiro, RJ, Brasil

**Keywords:** antifungal drug, minimum inhibitory concentration, Sporothrix brasiliensis

## Abstract

**BACKGROUND:**

*Sporothrix brasiliensis* is the most virulent sporotrichosis agent. This species usually responds to antifungal drugs, but therapeutic failure can occur in some patients. Antifungal susceptibility tests have been performed on this species, but no clinical breakpoints (CBPs) are available. In this situation, minimal inhibitory concentration (MIC) distributions and epidemiological cutoff values (ECVs) support the detection of identification of resistant strains.

**OBJECTIVES:**

To study the MIC distributions of five antifungal drugs against *S. brasiliensis* and to propose tentative ECVs.

**METHODS:**

MICs of amphotericin B (AMB), itraconazole (ITR), ketoconazole (KET), posaconazole (POS), and terbinafine (TRB) against 335 *S. brasiliensis* strains were determined by the Clinical and Laboratory Standards Institute broth microdilution method.

**FINDINGS:**

The proposed ECV, in µg/mL, for AMB, ITR, KET, POS, and TRB were 4.0, 2.0, 1.0, 2.0, and 0.25, respectively. Percentages of wild-type strains in our population for the above antifungal drugs were 98.48, 95.22, 95.33, 100, and 97.67%, respectively.

**MAIN CONCLUSIONS:**

These ECVs will be useful to detect strains with resistance, to define CBPs, and to elaborate specific therapeutic guidelines for *S. brasiliensis*. Rational use of antifungals is strongly recommended to avoid the emergence of resistant strains and ensure the therapeutic effectiveness of sporotrichosis.

*Sporothrix brasiliensis* is an emerging species of the *Sporothrix* complex, which is restricted to Brazil, and is highly pathogenic to humans and animals. This species is responsible for most sporotrichosis cases in this country, especially in the Southeast region, and is transmitted from infected cats to humans (Gutierrez-Galhardo et al. 2015). Studies using mouse models of experimental infection show that *S. brasiliensis* is highly virulent, resulting in high mortality rates in animals and a high fungal burden in several organs (Arrillaga-Moncrieff et al. 2009). In humans, this species is associated with some unusual manifestations of sporotrichosis, such as hypersensitivity reactions, disseminated infection in non-AIDS patients, central nervous system tropism, and ocular involvement (Almeida-Paes et al. 2014, Macedo et al. 2015).

Although this is a sympatric species (Gutierrez-Galhardo et al. 2015), sporotrichosis cases caused by *S. brasiliensis* can be found in other countries because of the migration of humans, the transport of pet cats, or the return of international travellers from endemic areas. When these imported mycoses are diagnosed in non-endemic areas, the lack of experience of clinicians and laboratory workers can delay diagnosis and make clinical management and treatment more difficult (Lelievre et al. 2015).

The first drug choice for sporotrichosis treatment is itraconazole (ITR), with terbinafine (TRB) and potassium iodide as alternatives; amphotericin B (AMB) is indicated in cases of severe disease (Kauffman et al. 2007). Most patients infected with *S. brasiliensis* respond well to these drugs (Francesconi et al. 2011, Almeida-Paes et al. 2014, Macedo et al. 2015), although therapeutic failures have been reported (Almeida-Paes et al. 2016). In order to guide therapy, antifungal susceptibility tests were developed (CLSI 2008). For some fungus-antifungal drug combinations, there are clinical breakpoints (CBPs). CBPs categorise strains that are inhibited by drug concentrations usually achievable at the site of infection as susceptible and strains that are not inhibited by achievable concentrations of the agent administered under normal dosage schedules as resistant. These CBPs are based on the minimal inhibitory concentration (MIC) distributions for each drug and species, the biochemical, molecular, and pharmacodynamic properties of the antifungal drugs, and the clinical data related to these drugs with these MIC values (Alastruey-Izquierdo et al. 2015). Although a reference method has been described for *Sporothrix schenckii* MIC determinations, there are no defined CBPs for any species that causes sporotrichosis (CLSI 2008). When CBPs are unavailable, the establishment of epidemiological cutoff values (ECVs) provides a means to detect strains with emerging resistance against antifungal drugs. Strains with MIC values equal or less than the ECVs are defined as wild-type (WT) strains, i.e., strains that does not harbour any acquired resistance to a tested antifungal agent. In contrast, strains with MICs higher than the ECVs have elevated odds to present acquired or mutational resistance mechanisms to the tested drug (Espinel-Ingroff & Turnidge 2016).

In the present study, a large collection of *S. brasiliensis* strains was tested against five different drugs to determine MIC distributions and to establish ECVs for each antifungal agent against this species.

## MATERIALS AND METHODS

*Strains* - A total of 335 *S. brasiliensis* strains obtained from 2002 to 2015 were included in this study. These strains were obtained from individual cases of human sporotrichosis (n = 212), feline sporotrichosis (n = 122), and the environment (n = 1). The Research Ethics Committee of Evandro Chagas National Institute of Infectious Diseases (INI)/Fiocruz approved this study under protocol number CAAE 26637014.8.0000.5262. The strains were identified as *S. brasiliensis* by partial sequencing of the calmodulin gene or T3B polymerase chain reaction (PCR) fingerprinting (Oliveira et al. 2012) and were stored at -20ºC until used. Strains were subcultured on potato dextrose agar (PDA) prior to antifungal testing.

*Antifungal drugs* - AMB, ITR, ketoconazole (KET), posaconazole (POS), and TRB were obtained from Sigma-Aldrich (St. Louis, MO). Two-fold serial dilutions of stock solutions of these antifungal agents prepared in dimethyl sulphoxide (DMSO) were performed to obtain working concentrations of antifungal drugs ranging from 0.015 to 8 mg/L, in a constant concentration of 1% DMSO.

*Antifungal susceptibility testing* - Inocula of 1-5 × 10^4^ conidia/mL were prepared in sterile saline solution after incubation of each strain in PDA for seven days at 37ºC. The broth microdilution reference method was performed according the M38-A2 CLSI guidelines (CLSI 2008), using RPMI 1640 medium buffered to pH 7.0 with 0.165 mol/L morpholinepropanesulfonic acid (Sigma-Aldrich). The different working concentrations of antifungal drugs were distributed into wells of round-bottom 96-well microplates. Wells containing RPMI-1640 medium with 1% DMSO and the fungal inoculum and without any antifungal agent were used as growth controls, or, when only medium with DMSO was added, as a sterility controls. Quality controls with the reference strains *Aspergillus fumigatus* ATCC 204305 and *Aspergillus flavus* ATCC 204304 were included in each microplate. Tests were validated only if MIC values for these strains were within the range described in the M38-A2 reference document. MICs were determined by visual inspection after 48-72 h of incubation at 35ºC, as described (CLSI 2008). In brief, for AMB, ITR, and POS, the MIC endpoints were the lowest concentrations that completely inhibited fungal growth. For KET, the MIC was the lowest concentration that resulted in a 50% reduction in growth relative to that of the growth control, and, for TRB, it was the lowest concentration that resulted in at least an 80% reduction in growth relative to the control without the antifungal drug. All strains were tested at least twice against AMB, KET, ITR, and TRB. POS was tested only with strains isolated from human patients.

*Analysis of results* - GraphPad Prism 5 software was used for data analysis. The range, MIC50, MIC90, and geometric means with a 95% confidence interval (CI) were obtained by a descriptive statistics analysis. Frequency log_2_ MIC distributions of each antifungal drug were plotted as histograms (Turnidge et al. 2006). To compare the in vitro efficacy of azoles against *S. brasiliensis*, ITR, KET, VOR, and POS MICs were compared using the non-parametric Mann-Whitney test. This test was also used to compare the antifungal susceptibilities of the antifungal agents against strains of human or animal origin. A p < 0.05 was considered statistically significant. ECVs were calculated as described previously (Espinel-Ingroff et al. 2014, Cafarchia et al. 2015), considering the modal MIC, the MIC distribution, the assumption that the WT population should cover three to five two-fold dilutions around the modal MIC, the assumption that an ECV should encompass at least 95% of WT organisms, and the inherent variability of the test, usually within one two-fold dilution. This variability can result from small lot-to-lot variations in media and reagents, the incubation atmosphere, imprecision in dilutions and inoculum preparation, and inaccuracies in endpoint visual determinations. Strains with MICs ≤ ECVs were classified as WT strains, and those with MICs > ECVs were classified as non-WT strains (Espinel-Ingroff & Turnidge 2016). The probability of a strain with a MIC above the ECV exhibiting resistance to the drug was calculated using the probability mass function as previously described (Turnidge et al. 2006).

## RESULTS

The MIC values of five antifungal drugs against *S. brasiliensis* are shown in Table I. In general, the MIC of TRB was at least two-fold lower than those observed for the other drugs, except for that the MIC of TRB was one-fold dilution lower than MIC of KET. Among the azoles, KET was the most effective antifungal drug (p < 0.0001), and the in vitro susceptibilities of *S. brasiliensis* to ITR and POS were similar to one another (p = 0.7116). Trailing end points for these agents and *S. brasiliensis* were not encountered, as reported before for *Aspergillus* and other fungi (CLSI 2008).

**TABLE I:** Minimal inhibitory concentrations (MIC) (µg/mL) of five antifungal drugs against *Sporothrix brasiliensis*

Antifungal drug	Range	GM (95% CI)	MIC50	MIC90
Amphotericin B	0.12 - 8.0	1.097 (1.014 - 1.188)	1.0	4.0
Itraconazole	0.06 - 16.0	0.762 (0.695 - 0.835)	1.0	2.0
Ketoconazole	0.03 - 2.0	0.356 (0.317 - 0.4)	0.5	1.0
Posaconazole	0.06 - 2.0	0.756 (0.695 - 0.823)	1.0	2.0
Terbinafine	0.015 - 1.0	0.072 (0.064 - 0.08)	0.06	0.25

CI: confidence interval; GM: geometric mean.

Table II presents a comparison of MICs of AMB, KET, ITR, and TRB against strains isolated from human and cats with sporotrichosis. In general, KET and ITR were associated with similar susceptibilities in strains from these two sources, whereas the MICs of AMB and TRB were higher for strains of human origin.

**TABLE II:** Comparison between minimal inhibitory concentrations (MIC) (µg/mL) of four antifungal agents against strains isolated from humans or cats with sporotrichosis

Antifungal	Human strains		Cat strains		p value
	
	Median	GM (95% CI)	Median	GM (95% CI)	
AMB	1.0	1.249 (1.131-1.378)	1.0	0.8822 (0.7796-0.9984)	< 0.0001
KET	0.25	0.3407 (0.2759-0.4207)	0.5	0.3742 (0.3282-0.4266)	0.2786
ITR	1.0	0.7798 (0.6818-0.8919)	1.0	0.7311 (0.6655-0.8033)	0.7750
TRB	0.12	0.0943 (0.0843-0.1055)	0.03	0.0369 (0.0317-0.0429)	< 0.0001

AMB: amphotericin B; CI: confidence interval; GM: geometric mean; ITR: itraconazole; KET: ketoconazole; TRB: terbinafine.

The MIC distributions for AMB, ITR, KET, POS, and TRB against *S. brasiliensis* are presented in Figure. The modal MIC values (in µg/mL) at 72 h of incubation for these drugs were 1.0, 1.0, 0.5, 1.0, and 0.125, respectively. The ECVs of *S. brasiliensis* were 4.0 µg/mL for AMB, 2.0 µg/mL for ITR, 1.0 µg/mL for KET, 2.0 µg/mL for POS, and 0.25 µg/mL for TRB. All tested strains were classified as WT when treated with POS. Non-WT strains were detected, in increasing order, with AMB, TRB, KET, and ITR (Table III). These strains were isolated throughout the years 2003 to 2014, i.e., there was no temporal clustering of non-WT strains. The probability of non-WT strains exhibiting resistance against the tested drugs ranged from 95.01-99.94%.

**Figure:**
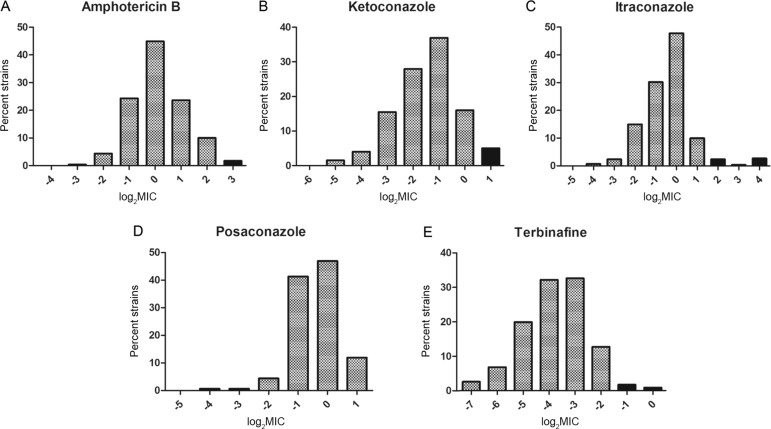
Minimal inhibitory concentration distribution of *Sporothrix brasiliensis* against amphotericin B (A), ketoconazole (B), itraconazole (C), posaconazole (D), and terbinafine (E), indicating wild-type strains (dotted bars) and non-wild-type strains (solid bars).

**TABLE III:** Proposed epidemiological cutoff values for five antifungal drugs against *Sporothrix brasiliensis*

Antifungal drug	ECV (µg/mL)		MIC (%) of non-WT isolates	Probability to harbor resistance

	WT	Non-WT		
Amphotericin B	≤ 4.0	≥ 8.0	8.0 µg/mL (1.52)	99.05%
Itraconazole	≤ 2.0	≥ 4.0	4.0 µg/mL (2.09)	95.01%
			8.0 µg/mL (0.30)	99.24%
			16.0 µg/mL (2.39)	99.94%
Ketoconazole	≤ 1.0	≥ 2.0	2.0 µg/mL (4.67)	95.6%
Posaconazole	≤ 2.0	≥ 4.0	-	-
Terbinafine	≤ 0.25	≥ 0.5	0.5 µg/mL (1.55)	97.39%
			1.0 µg/mL (0.78)	99.68%

ECV: epidemiological cutoff value; MIC: minimal inhibitory concentration; WT: wild-type.

## DISCUSSION

The MIC distributions for ITR, KET, POS, and TRB showed good susceptibility of *S. brasiliensis* to these drugs overall. The MIC distribution for AMB suggested moderate efficacy of this drug. Moreover, since the calculated ECV for this antifungal drug so high, it makes difficult to distinguish between wild type WT and non-wild WT type isolates. One possible weakness of this study was that MIC distributions were studied in strains isolated from a single centre. This limitation was mitigated by the fact that the isolates were obtained over seven years, different lots of broth microdilution reagents and drugs were used, and several qualified professionals read the MIC endpoints. This strategy was also used in a study performed in two centres to determine ECVs for unusual *Candida* species (Pfaller et al. 2011). Moreover, MIC ranges of other studies for *S. brasiliensis* antifungal susceptibility are within the MIC distributions observed in this study (Marimon et al. 2008, Ottonelli-Stopiglia et al. 2014, Rodrigues et al. 2014a, Borba-Santos et al. 2015, Almeida-Paes et al. 2016, Brilhante et al. 2016). It is also important to note that *S. brasiliensis* is currently found only in Brazil, and its major hyperendemic area is Rio de Janeiro state (Almeida-Paes et al. 2014, Gutierrez-Galhardo et al. 2015), where this study was performed.

CLSI reference method M38-A2 (CLSI 2008) is laborious and difficult to apply in most clinical laboratories, especially those with limited financial resources. One study showed very high agreement in *S. schenckii* ITR susceptibilities between the microdilution method and commercial tests such as Sensititre YeastOne and ATB Fungus (Alvarado-Ramirez & Torres-Rodriguez 2007). Therefore, we recommend that laboratories unable to perform the microdilution assay use commercial kits to determine *Sporothrix* susceptibilities to the most common antifungal agents used in the treatment of sporotrichosis. Further studies to determine the ECVs of antifungal drugs in *S. brasiliensis* with commercial tests are needed.

The ECVs of *S. brasiliensis* calculated in this study were similar to the ECVs of *Aspergillus* spp. (Espinel-Ingroff & Turnidge 2016), another filamentous genus described in the CLSI M38-A2 document (CLSI 2008). For fungi in the genus *Aspergillus*, ECVs are useful for determining whether an isolate harbours mutations in the *Cyp51A* gene (Espinel-Ingroff et al. 2010). Moreover, ECV are relevant in the use of POS in the treatment of an experimental mouse model of aspergillosis (Calvo et al. 2012). Despite the differences between these two organisms, the clinical pertinence of the *Aspergillus* ECV suggests that the ECVs proposed in this study for *S. brasiliensis* could also may be useful for epidemiologic and clinical purposes.

Most patients with sporotrichosis in Rio de Janeiro, Brazil, where *S. brasiliensis* predominates (Almeida-Paes et al. 2014, Gutierrez-Galhardo et al. 2015), have been treated with ITR and TRB (Francesconi et al. 2011, Almeida-Paes et al. 2016). To the best of our knowledge, POS has been used only once, to treat a single case of disseminated sporotrichosis that was refractory to AMB and ITR and that was caused by *S. brasiliensis* in a patient with AIDS, resulting in a clinical response and clearance of the fungus from the central nervous system (Paixão et al. 2015). However, this antifungal is seldom used because of its high cost and because the Brazilian government does not distribute this drug to treat mycotic infections. Oral KET has been described for the treatment of feline sporotrichosis, but it showed less effectiveness than oral ITR (Pereira et al. 2010). The similar susceptibilities to KET and ITR between cat and human strains in our study support the idea of clonal transmission of sporotrichosis between cats and humans in Rio de Janeiro (Rodrigues et al. 2014b). To the best of our knowledge, TRB was not used to treat domestic animals with sporotrichosis during the study period, and intralesional AMB was used only in association with oral ITR in cases refractory to this azole (Gremião et al. 2011).

Significant differences in antifungal susceptibilities have been observed among pathogenic *Sporothrix* spp. In general, *S. brasiliensis* shows a better response to antifungal drugs, especially ITR (Marimon et al. 2008, Rodrigues et al. 2014a). Therefore, the ECVs described here should only be used in *Sporothrix* strains identified as *S. brasiliensis* by molecular methods (Oliveira et al. 2012, Rodrigues et al. 2014a).

A few strains with higher probability of exhibiting resistance to the antifungal drugs used in the treatment of sporotrichosis were found in our study. The indiscriminate use of these antifungal drugs to treat classical and non-aggressive forms of sporotrichosis and other mycoses may have led to the emergence of resistance mechanisms. Sporotrichosis can be treated with non-antimycotic drugs such as potassium iodide (Bonifaz et al. 2007, Macedo et al. 2015) and with non-pharmacologic strategies such as hyperthermia (Bonifaz et al. 2007) and cryosurgery (Almeida-Paes et al. 2016), or even with a combination of low drug dosages and hyperthermia (Haruna et al. 2006). The non-pharmacologic approaches are interesting because they often accelerate clinical cures, reducing treatment costs. Therefore, we strongly recommend the rational use of antimycotic drugs to delay the emergence of resistant strains and to preserve these therapies for more complex sporotrichosis cases.

The detection of strains with elevated MICs against AMB, KET, ITR, and TRB suggests the emergence of *S. brasiliensis* strains that are resistant to these drugs. The molecular characterisation of *Sporothrix* strains is of increasing interest worldwide, and related techniques will make it possible to easily diagnose *S. brasiliensis* infections in countries other than Brazil in the future. These ECVs may help clinicians to appropriately manage sporotrichosis caused by *S. brasiliensis* and to monitor emerging resistance against antifungal drugs used to treat this mycotic infection. Because MICs and ECVs can vary according to several factors, including incubation time or medium composition, it is extremely important that these ECVs be used with the same experimental conditions described here. Given that we were not able to obtain clinical data associated with all strains included in this study, a multicentric validation, corroborating data from experimental models of infection, and clinical correlations with *Sporothrix* ECVs are needed to devise better strategies for sporotrichosis treatment.
